# Determinants of maternal health service utilization in Ethiopia: analysis of the 2011 Ethiopian Demographic and Health Survey

**DOI:** 10.1186/1471-2393-14-161

**Published:** 2014-05-07

**Authors:** Shegaw Mulu Tarekegn, Leslie Sue Lieberman, Vincentas Giedraitis

**Affiliations:** 1 Department of Health Management Information Systems, Tulane International, Addis Ababa, Ethiopia; 2 Department of Anthropology, University of Florida, Orlando, FL 32816-0955, USA; 3 Faculty of Economics, Vilnius University, Vilnius, Lithuania

**Keywords:** Antenatal care, Delivery, Postnatal care, Maternal health service, Determinants, Skilled delivery attendant, Ethiopia

## Abstract

**Background:**

Antenatal Care (ANC), use of skilled delivery attendants and postnatal care (PNC) services are key maternal health services that can significantly reduce maternal mortality. Understanding the factors that affect service utilization helps to design appropriate strategies and policies towards improvement of service utilization and thereby reduce maternal mortality. The objective of this study was to identify factors that affect utilization of maternal health services in Ethiopia.

**Methods:**

Data were drawn from the 2011 *Ethiopia Demographic and Health Survey*. The dependent variables were use of ANC, skilled delivery attendants and PNC services. The independent variables were categorized as socio-cultural, perceived needs and accessibility related factors. Data analysis was done using SPSS for windows version 20.0. Bivariate and multivariate logistic regression models were used in the analysis.

**Results:**

Thirty four percent of women had ANC visits, 11.7% used skilled delivery attendants and 9.7% of women had a postnatal health checkup. Education of women, place of residence, ethnicity, parity, women’s autonomy and household wealth had a significant association with the use of maternal health services. Women who completed higher education were more likely to use ANC (AOR = 3.8, 95% CI = 1.8-7.8), skilled delivery attendants (AOR = 3.4, 95% CI = 1.9-6.2) and PNC (AOR = 3.2, 95% CI = 2.0-5.2). Women from urban areas use ANC (AOR = 2.3, 95% CI = 1.9-2.9), skilled delivery attendants (AOR = 4.9, 95% CI = 3.8-6.3) and PNC services (AOR = 2.6, 95% CI = 2.0-3.4) more than women from rural areas. Women who have had ANC visits during the index pregnancy were more likely to subsequently use skilled delivery attendants (AOR = 1.3, 95% CI = 1.1-1.7) and PNC (AOR = 3.4, 95% CI = 2.8-4.1). Utilization of ANC, delivery and PNC services is more among more autonomous women than those whose spending is controlled by other people.

**Conclusion:**

Maternal health service utilization in Ethiopia is very low. Socio-demographic and accessibility related factors are major determinants of service utilization. There is a high inequality in service utilization among women with differences in education, household wealth, autonomy and residence. ANC is an important entry point for subsequent use of delivery and PNC services. Strategies that aim improving maternal health service utilization should target improvement of education, economic status and empowerment of women.

## Background

Worldwide, approximately 800 women die every day from preventable causes related to pregnancy and childbirth. In 2010, about 287,000 women died worldwide during and following pregnancy and childbirth [1,2]. Though this is a decline of 47% from the 1990 level, it is still far from the 2015 Millennium Development Goal (MDG). The fifth MDG calls for a reduction in the maternal mortality ratio by 75% between 1990 and 2015. The key indicators to measure this goal are the proportion of pregnant mothers who received ANC and the proportion of births attended by skilled delivery attendants [1,3].

Despite proven interventions that could prevent death or disability during pregnancy and childbirth, maternal mortality remains a major burden in many developing countries. Maternal mortality continues to be a major challenge in Africa and the maternal mortality disparity between developing and developed countries is very high. The maternal mortality ratio (MMR) in developing regions is 15 times higher than in the developed regions [1,3,4] and sub Saharan African countries have the highest MMR in the world with an average of 500 maternal deaths per 100,000 live births, accounting for half of the world’s total maternal deaths [1,2,5]. Most women die because they give birth without the attendance of a skilled health worker [1,2].

Ethiopia is one of the countries with high maternal mortality. The MMR was 871 per 100,000 in the year 2000; it was 673 per 100,000 live births in 2005 and 676 per 100,000 in 2011. Maternal deaths represent 30% of all deaths to women age 15–49, compared with 21% in the 2005 EDHS and 25% in the 2000 EDHS [6-8].

Evidence shows that high maternal, neonatal and child mortality rates are associated with inadequate and poor-quality maternal health care [9]. Moreover, evidences also show that killed care before, during and after childbirth saves the lives of women and newborn babies. An estimated 74% of maternal deaths could be averted if all women had access to the interventions for preventing or treating pregnancy and birth complications, in particular emergency obstetric care [10]. As a result, the use of ANC, skilled delivery attendants and PNC are recognized as key maternal health services to improve health outcomes for women and children [1,9].

The antenatal period is critically important for reaching women with interventions and information that promote health, wellbeing and survival of mothers as well as their babies. The coverage of at least one visit with a doctor, nurse or midwife has progressively increased in developing regions from 63% in 1990 to 71% in 2000, and then to 80% in 2010. In Ethiopia, according to the EDHS reports, the percentage of women with at least one ANC visit by a health professional was only 28% in 2005 and 33% in 2011 [7,8]. In Ethiopia, Only 19% had 4 ANC visits as recommended by the WHO [8].

Sub-Saharan Africa is the region with the lowest coverage of skilled delivery utilization, with only 45% of women having skilled delivery attendants [3]. In Ethiopia, skilled delivery utilization is very low with only 10% of women having delivered with an assistance of a skilled delivery attendant. The PNC utilization in Ethiopia is also very low, with only 7% of women having postnatal care service in the first 2 days after delivery [8].

The objectives of this research are:

1. To determine the level of utilization of ANC, skilled delivery attendants and PNC services in Ethiopia.

2. To identify factors that affect the utilization of ANC, use of skilled delivery attendants and PNC services in Ethiopia.

The research answers the following questions related with utilization of maternal health services in Ethiopia.

1. What is the level of utilization of ANC, delivery and PNC service in Ethiopia?

2. Which factors (such as age, education, parity, ethnicity, religion, geographic location) are related to maternal health service utilization in Ethiopia?"

Understanding the factors that affect the utilization of these important maternal health services can help design strategies and develop policies toward improvement of service utilization in the country; and thereby, will aid in decreasing maternal mortality.

## Method**s**

This study utilized secondary data from the 2011 Ethiopian Demographic and Health Survey (EDHS). The data are thoroughly analyzed using bivariate and multivariate logistic regression. The survey data were downloaded from Measure DHS website after data use permission was guaranteed. The 2011 EDHS is part of the worldwide MEASURE DHS project which was funded by the United States Agency for International Development (USAID) and was implemented by the Ethiopian Central Statistical Agency. A DHS is undertaken every 5 years and the 2011 survey is the third DHS in Ethiopia. The first DHS was performed in 2000 and the second was performed in 2005 [11].

### Study design

It is a community based analytical cross sectional study. The data was collected from a representative sample of women in the reproductive age group (age 15-49) from all regions in Ethiopia. The analysis is based on data from women who had at least one birth during the 5 years preceding the survey (8).

### Study population

**Source population:** The source population for this study was all women who are in the reproductive age group (aged 15 to 49 years).

**Study subjects:** The study subjects were women aged 15 to 49 years who gave at least one birth in the last 5 years preceding the survey.

### Sample size

A national representative sample of 17,817 households was selected for the study. From these households, a total of 16,515 women in the reproductive age group were interviewed using a structured questionnaire (8). For this study, women who had at least one birth in the last five years preceding the survey were included in the analysis.

### Sampling procedures

A stratified, two stage cluster sampling procedure was used to identify the representative samples. The sampling frame for the 2011 EDHS consists of a total of 85,057 Enumeration Areas (EAs). An EA is a geographic area consisting of a convenient number of dwelling units. On the first stage, 624 EAs were selected from the total EAs using probability to proportional size method. Then, on the second stage, a fixed number of 30 households were selected from each EA. A total of 17,817 households were included in the interview (8).

The sampling frame excluded some special EAs with disputed boundaries. These EAs represent only 0.1% of the total population. In Somali region, all the listed households were not included in the interview due to drought and security reasons which makes the data for the region not representative. However, the national sample will not be affected because of the small proportion of the region’s population (8).

### Data collection procedures

A structured and pre-tested questionnaire was used as a tool for data collection. The questionnaire was developed in English and then translated into three different local languages (Amharic, Oromiffa and Tigrigna). The questionnaire was developed based on standard DHS survey questionnaires. Structured interview schedules were performed by trained interviewers. In order to maintain the quality of data to be collected, interviewers were trained, a pretest was performed before the actual data collection, frequent supervision was performed during data collection and interviews were performed using local languages (8).

### Operational definitions

**Skilled attendants:** Professionals who have midwifery skills including doctors, midwifes and nurses.

**ANC by skilled attendants:** Pregnancy care provided by skilled health professionals (doctors, midwives or nurses) during the respondent’s recent pregnancy.

**Use of skilled delivery attendants:** Delivery care provided by skilled health professionals (doctors, midwives or nurses) during the respondent’s recent birth.

**Postnatal care:** Care provided to women within 42 days after delivery.

**Woman’s autonomy:** A more autonomous woman is a woman who can decide on health care spending alone or with her husband. If the decision of health care spending is controlled by others (husband only or other people), it is considered as non-autonomous.

### Data analysis procedures

Analysis was done using SPSS version 20.0. Bivariate and multivariate analysis techniques were used during analysis. Frequencies were first determined followed by cross tabulations to compare frequencies. At bivariate level, analysis was made by the chi square (X^2^) test for categorical variables. The association between dependent and independent variables was measured by means of odds ratio for which 95% confidence interval was calculated. Variables that show a statistically significant association (p < 0.05) at bivariate level were further analyzed at multivariate level by logistic regression. All the variables were included in the multivariate model once they were significantly associated at the bivariate level. This is because these variables showed an influence on the outcome variable and there is a need to identify whether each has been confounded by another variable or not. The adjusted odds ratio (AOR) was used to determine the presence of association between the dependent and independent variables for which 95% CI was determined.

Whenever there is a non-proportional allocation of samples, use of sample weights is an important step during analysis [12]. During EDHS 2011, there was some non-proportional allocation of the samples to the different regions and their urban and rural areas in order to compensate for places with very low family planning coverage and low fertility areas. In order to ensure the actual representativeness of survey results at national level, sampling weights are used during the analysis.

The sampling used during the EDHS 2011 is a two-stage stratified cluster sample methodology. As a result, sample weights were calculated based on sampling probabilities separately for each sampling stage and for each cluster. The first stage is sampling probability of each cluster in each stratum and the second stage comprises of sampling probability within each cluster (households selected). Computation of the sampling probability for clusters and households is computed based on what the EDHS have computed. The complete sample weight computation is available with the data set that we get from measure evaluation (8).

### Conceptual framework and variables of the study

**Figure 1:**
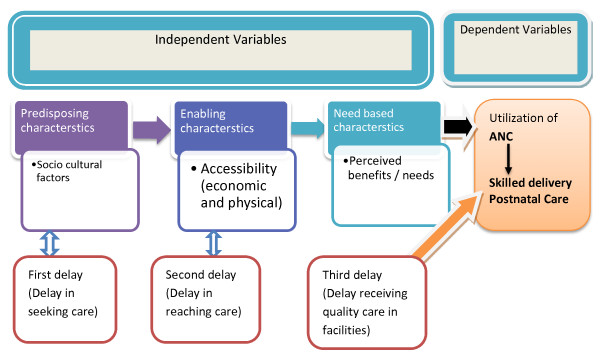
**Conceptual framework of the study.** Shows the conceptual framework of the study and is prepared based on Anderson’s behavioral model of the determinants of health service utilization and the three delays model of the determinant of maternal health services utilization. The figure shows the relationship between the independent variables and how it affects the dependent variables of the study.

The conceptual framework for this study was developed based on Andersen’s behavioral model and the three delays model of maternal health care utilization [13,14]. The conceptual framework of the study is shown on Figure 1.

Andersen’s behavioral model examines the influence of individuals' demographic characteristics and health delivery system variables on utilization patterns. It hypothesizes that the decision to seek medical help is a function of three sets of variables:

i. Predisposing factors, such as age, sex, marital status, family size, social status, education and ethnicity/race.

ii. Enabling factors: includes the logistical aspects of obtaining care which includes family income, health insurance, service availability and travel.

iii. The need to use service factor: perceived need, i.e. "How people view their own general health and functional state, as well as how they experience symptoms of illness, pain, and worries about their health and whether or not they judge their problems to be of sufficient importance and magnitude to seek professional help" [13].

The three delays model identifies three groups of factors which may stop women and girls accessing the levels of maternal health care that they need: These are

Phase 1: Delay in decision to seek care: mainly socio-demographic factors

Phase 2: Delay in reaching care: related with factors of physical and economical accessibility

Phase 3: Delay in receiving adequate health care: factors related with quality of health care in facilities [14].

### Ethical consideration

Ethical clearance for the survey (EDHS 2011) was provided by the Ethiopian Health and Nutrition Research Institute (EHNRI) Review Board, the National Research Ethics Review Committee (NRERC) at the Ministry of Science and Technology, the Institutional Review Board of ICF International, and the CDC. Respondents were informed about the survey and consent was taken for their participation. Voluntary participation was ensured during interviews (8). The researcher has received the survey data from Measure DHS upon submission of a proposal. After data access is authorized from Measure DHS, the researcher of this study has maintained the confidentiality of the data.

### Limitations of the study

• Data related to service availability and quality of health services were not collected. As a result, health facility related factors, which are the causes for the third delay in maternal mortality, were not analyzed.

• Recall bias: Women might have difficulty in remembering things that have happened during the last 5 years preceding the survey. Women may also have difficulty remembering or identifying the type of health professional who provided the service.

## Results

The results of the study are presented based on a descriptive, bivariate and multivariate logistic regression analysis. First, description of the study subjects was done followed by crude odds ratio analysis at bivariate level. Then, adjusted OR (AOR) was determined by a multivariate analysis. Factors that determine utilization of ANC, skilled delivery attendance and PNC services were organized into three categories as socio-cultural, accessibility and perceived need factors.

### Socio-demographic characterstics of the study subjects

A total of 7,908 women aged 15 to 49 years of age who had at least one birth five years before the survey were interviewed. The majority (85%) of the respondents were rural residents. Most of the respondents (91%) were either married or lived with a partner and only 0.9% were never married. Oromo and Amhara ethnic groups were the predominant ethnic groups accounting for 35% and 28.5% respectively. The majority of women (69.3%) were between the age 20 to 34 and 5.1% were aged 15 to 19 years of age. Regarding the level of education, 67% did not have any formal education. Orthodox Christians and Muslims constitute 42% and 32% of the total respectively. The household wealth was distributed similarly among the respondents. Forty-two percent of the study subjects were in the two poor wealth quintiles, 20.6% were in the middle and 36% were in the two upper wealth quintiles. Regarding the total number of children, 18% of them had only one child, 43.8% had 2 to 4 children and 38% had 5 or more children. The socio-demographic description of the study subjects is shown on Table 1.

**Table 1:** Socio-demographic characteristics of women who had at least one birth in the five years preceding the survey

**Background Characteristics**	**Number**	**Percent**
Residence		
Urban	1188	15.0
Rural	6720	85.0
Age		
15-19	402	5.1
20-34	5480	69.3
35-49	2026	25.6
Marital status		
Never Married	72	0.9
Married/living together	7185	90.9
Divorced/separated/widowed	651	8.2
Religion		
Orthodox	3327	42.1
Catholics	84	1.0
Protestants	1763	22.3
Muslims	2563	32.4
Others	175	2.2
Ethnicity		
Amhara	2257	28.5
Guragie	180	2.3
Oromo	2765	35.0
Sidama	334	4.2
Tigrie	524	6.6
Wolaita	213	2.7
Others	1635	20.7
Educational status		
No education	5270	66.6
Primary	2270	28.7
Secondary	226	2.9
Higher	142	1.8
Household Wealth		
Poorest	1739	22.0
Poorer	1696	21.5
Middle	1628	20.6
Richer	1494	18.9
Richest	1351	17.1
Parity		
1	1399	17.7
2-4	3464	43.8
5 or more	3045	38.5
No. of births in the last 5 yrs		
1	4505	57.0
2+	3403	43.0
Total (n)	**7908**	**100**

### Pattern of ANC, skilled delivery and PNC service utilization

Fifty-seven percent of women had no ANC visits while 42.9% had at least one ANC visit during their last pregnancy. The percentage of women who have had ANC by a skilled ANC attendant was only 33.9%. In this study, only 19.1% of women had four or more ANC visits during their last pregnancy. Only 26.2% of those who had ANC visits started their ANC visit during the recommended timing, i.e. during the first trimester of their pregnancy; the majority (56.4%) started during the second trimester and 16.7% started during the third trimester.

Eighty-eight percent of women delivered at home and 11.7% delivered in a health facility. No woman delivered at home with the assistance of a skilled attendant. Only 9.3% of women had a health checkup within six weeks after delivery. Table 2 shows the number and percentage of women who used ANC, skilled delivery attendants and PNC services.

**Table 2:** Number and percentage of women who had ANC visit, delivery and PNC services by women who had at least one birth in the five years preceding the survey

**Variables**	**Number**	**Percent**
Had at least one ANC		
Yes, by skilled provider	2674	33.9
No ANC	4507	57.0
Missing	9	0.1
Frequency of ANC during pregnancy		
None	4517	57.1
Once	352	4.5
2 times	522	6.6
3 times	982	12.4
4 or more	1508	19.1
Donot know	27	.3
Timing of first ANC		
Less than 4 months	888	26.2
4 to 6 months	1914	56.4
6-9 months	566	16.7
Do not know	24	.7
Total	3391	100.0
Used skilled delivery attendant		
Yes	932	11.7
No	6971	88.3
Place of delivery		
Home	6948	87.9
Facility	928	11.7
Other	29	.4
Missing	3	.0
Post natal care		
Yes	737	9.3
No	7159	90.7

### Bivariate analysis of the use of ANC, delivery and PNC services

The use of ANC, skilled delivery attendants and PNC services by background characteristics of respondents is shown on Table 3 below. The bivariate analysis shows the effect of each single independent variable on the utilization of ANC, skilled delivery attendants and PNC services.

**Table 3:** Percentage of women who had at least one birth in the five years preceding the survey who received skilled ANC, delivery and PNC service, by background characteristics

**Background characteristics**	**Percentage who received ANC at least once**	**Percentage who received delivery care**	**Percentage who received PNC care**
**Residence**			
Urban	76.1	53.7	33.7
Rural	26.4	4.4	5.0
Total	33.9	11.7	9.3
Marital status			
Never Married	36.4	28.1	19.1
Married/living together	33.8	11.3	8.9
Divorced/separated/widowed	33.9	15.8	12.6
Age			
15-19	32	9.8	9.4
20-34	36.0	13.8	10.3
35-49	28.6	6.8	6.8
Religion			
Orthodox	40.1	17.2	12.9
Catholics	36.2	14.1	10.3
Protestants	31.5	8.5	7.8
Muslims	28.1	7.7	6.2
Others	22.1	1.2	2.6
Ethnicity			
Amhara	38.7	17.3	10.8
Guragie	66.2	41.6	24.7
Oromo	32.7	9.8	7.5
Sidama	22.5	3.0	6.7
Tigrie	51.2	14.6	17.0
Wolaita	25.3	5.8	8.0
Others	23.5	6.0	6.9
Educational status			
No education	25.1	4.7	4.9
Primary	45.6	18.4	12.4
Secondary	85.5	72.8	50.7
Higher	90.0	73.6	57.4
HH Wealth			
Poorest	17.0	2.0	3.5
Poorer	23.8	3.0	5.2
Middle	26.8	3.4	3.0
Richer	35.6	8.0	6.9
Richest	74.9	49.6	32.4
Parity			
1	46.0	25.9	19.2
2-4	35.5	12.5	8.7
5 or more	26.4	4.5	5.5
No. of births in the last 5 yrs			
1	39.6	16.5	12.4
2+	26.3	5.6	5.3

#### Use of ANC service

Use of skilled ANC attendants is more common among residents of urban than rural locations. Seventy six percent (76%) of urban residents used skilled ANC attendants compared with only 26% of the rural women. With regard to marital status, it is slightly higher among never married women than other categories. Gurage ethnic groups have the highest proportion of women who use skilled ANC care: 66% of Guragie women use skilled ANC attendants compared with 38% of Amharas, 51% of Tigres and 32.7% of Oromo women. Skilled ANC utilization increases as the level of education of women increases. Only 25% of women who had no education used skilled ANC attendants compared with 45.5% of those with primary education, 85.6% of those with highschool education and 90% of those who had higher than secondary level education. Orthodox Christians have the highest proportion of women who use skilled ANC attendants with 40% of Orthodox Christians using skilled ANC attendants. As the household wealth quintile increases, the proportion of use of skilled ANC attendants also increased. Women from the richest households have the highest proportion of ANC attendance (75%) compared with 17% of those from the poorest household wealth quintile.

#### Use of skilled delivery attendants

The proportion of women who had used skilled delivery attendants is much higher (53%) among urban residents than those who are from the rural areas (4.4%). Never married women, Orthodox Christians, Guragie ethnic groups, those from the richest household, better educated women and those with only 1 birth had a higher proportion of using skilled delivery attendants (see Table 3). Utilization increases consistently as the educational level and the household wealth increases.

#### Use of postnatal care (PNC)

The use of PNC service is similar to the use of skilled delivery attendants. The proportion of women who had PNC was higher (33.5%) among urban residents than rural residents (5%). Never married women, Orthodox Christians, Guragie ethnic groups, those from the richest household, better educated women and those with only 1 birth had a higher proportion to use postnatal care services (see Table 3).

### Multivariate analysis of factors affecting the use of ANC, delivery and PNC services

During the multivariate analysis, a dichotomous logistic regression was employed. The dependent variables were categorized as use of ANC, skilled delivery attendants and PNC services. All variables that showed significant association during the bivariate analysis were included in the multivariate model. The P values, adjusted odds ratios (AOR) and 95% confidence intervals (CI) are presented in Table 4 below.

**Table 4:** Adjusted odds ratios and 95 percent confidence intervals for receiving ANC, delivery and PNC services

**Variables**	**Use of skilled ANC attendants**			**Use of skilled delivery attendants**			**Use of PNC**		
	Adjusted OR* (95% CI)		P value**	Adjusted OR* (95% CI)		P Value**	Adjusted OR* (95% CI)		P Value**
**Residence**									
Urban	2.3	1.9-2.9	0.001	4.9	3.8-6.3	0.001	2.6	2.0-3.4	0.001
Rural	1.0								
**Marital status**									
Never Married	1.3	1.1-1.6	0.008	0.8	0.4-0.9	0.027	1.8	1.1-3.2	0.035
Married/living together	0.9	0.8-1.1	0.154	0.7	0.5-0.8	0.001	0.6	0.5-0.8	0.001
Divorced/separated/widowed	1.0			1.0			1.0		
**Age**									
15-19	0.8	0.6-1.2	0.448	0.5	0.3-0.9	0.013	0.8	0.6-1.4	0.621
20-34	1.0	0.9-1.2	0.635	0.9	0.7-1.3	0.710	0.9	0.7-1.1	0.190
35-49	1.0			1.0			1.0		
**Religion**									
Orthodox	1.3	0.8-2.1	0.418	5.9	1.3-27.3	0.022	8.7	1.2-64	0.034
Catholics	2.2	0.8-3.5	0.183	5.5	0.9-31.9	0.057	3.4	0.4-31.3	0.284
Protestants	1.7	0.9-2.7	0.068	4.8	1.1-22.2	0.043	7.7	1.1-56.4	0.045
Muslims	1.5	0.8-2.2	0.258	4.2	0.9-18.9	0.066	7.2	1.0-53.0	0.051
Others	1.0			1.0		0.019			0.056
**Ethnicity**									
Amhara	1.9	1.4-2.1	0.001	1.1	0.8-1.5	0.541	0.8	0.6-1.1	0.088
Guragie	3.1	2.2-5.4	0.001	2.8	1.8-4.4	0.001	1.1	0.7-1.5	0.806
Oromo	1.2	1.1-1.4	0.038	1.2	0.9-1.5	0.206	0.8	0.7-1.1	0.142
Sidama	0.6	0.5-1.0	0.067	0.3	0.1-0.9	0.027	1.0	0.5-1.9	0.958
Tigrie	2.7	1.9-3.1	0.001	0.5	0.3-0.7	0.001	0.9	0.6-1.2	0.433
Wolaita	0.4	0.3-0.7	0.001	0.2	0.1-0.5	0.001	0.6	0.3-1.9	0.135
Others	1.0			1.0		0.001			0.374
**Educational status**									
No education	1.0					0.001			
Primary	1.6	1.4-1.9	0.001	1.6	1.3-2.0	0.001	1.3	1.1-1.6	0.026
Secondary	3.4	2.9-5.4	0.001	3.1	2.0-4.7	0.001	2.4	1.6-3.4	0.000
Higher	3.8	1.8-7.8	0.001	3.4	1.9-6.2	0.001	3.2	2.0-5.2	0.000
**HH Wealth**									
Poorest	1.0		0.001			0.001			0.001
Poorer	1.2	1.1-1.5	0.041	1.0	0.6-1.4	0.794	1.3	0.9-1.8	0.167
Middle	1.5	1.2-1.8	0.001	0.9	0.6-1.4	0.679	1.1	0.8-1.6	0.475
Richer	1.7	1.4-2.1	0.001	1.4	1.0-2.0	0.051	1.9	1.3-2.5	0.000
Richest	3.7	2.9-4.8	0.001	3.0	2.1-4.2	0.001	2.4	1.7-3.5	0.000
**Parity**									
1	1.2	0.9-1.5	0.138	2.4	1.7-3.4	0.001	1.4	1.1-1.9	0.019
2-4	1.1	1.0-1.3	0.169	1.4	1.1-1.8	0.017	1.1	0.8-1.3	0.702
5 or more						0.001			0.016
**No. of births in the last 5 yrs**									
1	1.3	1.1-1.5	0.001	1.3	1.0-1.6	0.025	-	-	-
2+	1.0			1.0					
**Husbands education**						0.001			
No	1.0								
Primary	1.3	1.1-1.5	0.001	1.4	1.1-1.7	0.009	1.1	0.8-1.3	0.624
Secondary	2.0	1.5-2.7	0.001	2.1	1.6-2.9	0.001	1.7	1.3-2.3	0.001
Higher	1.9	1.3-2.7	0.001	1.9	1.3-2.9	0.002	1.6	1.1-2.4	0.009
**Autonomy of woman on health care spending**									
Women + -Husband	1.4	1.2-1.6	0.001	1.3	1.1-1.7	0.022	1.0	0.8-1.2	0.853
Husband only or others	1.0			1.0					
**Husband’s work status**									
Jobless	1.0								
working	1.1	1.1-1.3	0.045	-	-	-	-	-	-
**Woman’s work status**									
Jobless							-	-	-
working	1.1	1.1-1.3	0.033	0.9	0.8-1.1	0.576	-	-	-
**Reading newspaper frequency**									
Not at all	1.0								
Less than once a week	1.1	0.8-1.5	0.375	1.2	0.9-1.6	0.187	1.0	0.7-1.3	0.802
At least once a week	0.9	0.5-1.6	0.726	2.1	1.1-3.7	0.016	1.1	0.7-1.8	0.548
**Listening radio frequency**									
Not at all	1.0								
Less than once a week	1.4	1.2-1.6	0.001	0.9	0.7-1.1	0.418	1.0	0.8-1.2	0.778
At least once a week	1.3	1.1-1.6	0.002	0.9	0.8-1.2	0.774	1.2	1.0-1.5	0.099
**Watching television frequency**									
Not at all	1.0								
Less than once a week	1.3	1.1-1.5	0.001			-	-	-	-
At least once a week	1.3	1.3-2.0	0.001		-	-	-	-	-
**USE OF ANC during the index pregnancy**									
Yes	-	-	-	1.3	1.1-1.7	0.001	3.4	2.8-4.1	0.001
No	-	-	-	1.0	-	-	1.0	-	-

*The AORs have been adjusted for all other variables in the model.

**The statistical test used was Chi square test. A 95%CI used to determine significant association.

#### Socio-cultural factors and the utilization of maternal health services

##### Religion

**Use of skilled ANC attendants**: There was no significant association between religion and use of skilled ANC attendants.

**Use of skilled delivery attendants**: Utilization of skilled delivery attendants was significantly associated with religion of women. Orthodox Christians (AOR = 5.9, 95% CI = 1.3-27.3) and Protestants (AOR = 4.8, 95% CI = 1.1-22.2) were more likely to use skilled delivery attendants compared to women of other religions. However, no significant association was found between two religious denominations, i.e Catholics and Muslims.

**Use of postnatal care**: There was no significant association between religion and use of postnatal care service.

##### Ethnicity

**Use of skilled ANC attendants**: Ethnicity was significantly associated with the use of skilled ANC attendants. Guragie ethnic groups were more likely to use skilled ANC attendants than other ethnic groups (AOR = 3.1, 95% CI, 2.2-5.4). On the other hand, Wolaita ethnic groups were less likely to use skilled ANC service (AOR = 0.4, 95% CI = 0.3-0.7).

**Use of skilled delivery attendants**: Ethnicity was found to be significantly associated with use of skilled delivery attendants. Guragie, Amhara, Oromo and Tigre ethnic groups were more likely to use skilled delivery attendance but Wolaita ethnic groups were less likely to use skilled delivery attendants.

**Use of postnatal care**: There was no significant association between ethnicity and the use of postnatal care service.

##### Education of women and their husbands/partners

**Use of skilled ANC attendants**: Both education of women and their husbands was found to have a significant association with the use of skilled ANC attendants. Women who have completed secondary school and higher education were more likely to use skilled ANC attendants than women who had no education (for higher education, AOR = 3.8, 95% CI = 1.8-7.8). Women whose husbands were educated to secondary and higher education level were also more likely than women whose husbands/partners were not educated (for higher education, AOR = 1.9, 95% CI = 1.3-2.7).

**Use of skilled delivery attendants:** Use of skilled delivery attendants was significantly associated with both education of women and their husbands. As the education level of women and their husbands increased, the likelihood of using skilled delivery attendants also increased. Women who are educated to high school level and higher were more likely to use skilled delivery attendants (AOR = 3.4, 95% CI = 1.9-6.2) than those who were not educated. Women whose husbands were educated to high school level and higher were also more likely to use skilled delivery attendants.

**Use of postnatal care:** A similar finding to the use of ANC and skilled attendants was found between education level and use of PNC service. The likelihood of women to use PNC service was high among women who had a higher educational level (AOR = 3.2, 95% CI = 2.0-5.2).

##### 

**Maternal age and parity** The multivariate analysis shows that maternal age was not associated with the use of all the three maternal health services. However, parity was found to have a significant effect on the use of the three maternal health services. The effect of age on service utilization is absent after controlling for parity of women during the multivariate analysis.

**Use of skilled ANC attendants:** The number of births in the last 5 years before the survey was significantly association with use of skilled ANC service. Women who had only one birth during the last five years were more likely to use ANC service (AOR = 1.3, 95% CI = 1.1-1.5) than those who had two or more births.

**Use of skilled delivery attendants**: Parity was also found to have a significant association with the use of skilled delivery attendants. Mothers who had 1 births (AOR = 2.4, 95% CI = 1.7-3.4) and 2-4 births (AOR = 1.4, 95% CI = 1.1-1.8) were more likely to use skilled delivery attendants respectively.

**Use of postnatal care:** Women with only one birth were more likely to use PNC services than those who had 5 or more births (AOR = 1.4, 95% CI = 1.1-1.9).

##### 

**Marital status** Never married women were found to be more likely to use skilled ANC attendants (AOR = 1.3, 95% CI = 1.1-1.6) and PNC services than others (AOR = 1.8, 95% CI = 1.1-3.2). Married women were less likely to use ANC attendants and PNC services. With regards to the use of skilled delivery attendants, never married women (AOR = 0.8, 95% CI = 0.4-0.9) and married women (AOR = 0.5, 95% CI = 0.5-0.8) were found to be less likely to use skilled delivery attendance than divorced/separated/widowed women.

##### 

**Autonomy of women on health care spending** Autonomy of women on health care spending was found to have a significant association with the utilization of skilled ANC and delivery attendants. Autonomous women were more likely to use ANC (AOR = 1.4, 95% CI = 1.2-1.6) and delivery attendants (AOR = 1.3, 95% CI = 1.1-1.7).

#### 

##### Factors related with perceived benefits and the utilization of maternal health services

**Pregnancy wantedness** Women were asked whether their last pregnancy was wanted or not. Eleven percent of women (N = 858) had their last pregnancy unwanted and 89.1% (N = 7047) of women had their last pregancy wanted. Wantedness of the index pregnancy did not have any significant association with the use of ANC, skilled delivery attendants and PNC services [Crude OR = 0.9, 95% CI = 0.8-1.1 for ANC and Crude OR = 1.0, 95% CI = 0.8-1.2 for the use of skilled delivery attendants; Crude OR = 0.9, 95% CI = [0.7-1.1] for PNC].

##### 

**Use of public information sources** Reading newspapers did not have any association with the utilization of maternal health services. Before controlling for other variables at bivariate level of analysis, it was significantly associated with use of ANC (Crude OR 6.6, 95% CI = 5.4-8.0). The effect is absent after controlling for other variables like education and residence of the respondent. The reason for the absence of association on multivariate analysis is because of the confounding effect of the other variables.

Listening radio programs and frequency of television watching have had a significant association with the utilization of ANC services. Women who listen to radio programs were more likely to use maternal health services than those who never listen (AOR = 1.3, 95% CI = 1.1-1.6). The effect of watching television was also similar to listening to radio programs. However, there was no association between listening radio and watching television programs with the use of delivery and PNC services.

##### 

**Effect of ANC use on use of skilled delivery attendants** Women who had ANC visits during the index pregnancy were more likely to use skilled delivery attendants than those who did not have ANC follow up. Women who have had ANC during the index pregnancy were more likely to use skilled delivery attendants (AOR = 1.3, 95% CI = 1.1-1.7). Moreover, women who had attended ANC visits during the index pregnancy were also more likely to attend PNC services (AOR = 3.4, 95% CI = 2.8-4.1).

#### 

##### Accessibility related factors and the use of maternal health services

**Residence** Women’s place of residence was significantly associated with use of skilled ANC services. Women in urban residence were more likely to use skilled ANC attendants (AOR = 2.3, 95% CI = 1.9-2.9). Regarding the use of skilled delivery attendants and the use of PNC services, a similar result was found. Women who were from urban residence were more likely to use skilled delivery attendants than those who were from rural residence (AOR = 4.9, 95% CI = 3.8-6.3). Use of PNC service was also more likely among urban residents compared with rural residents (AOR = 2.6, 95% CI = 2.0-3.4).

##### 

**Work status of women and their husbands/partners** Work status of husbands did not have a significant association with the use of all the three maternal health services. However, work status of women had a significant association with the use of skilled ANC services. Women who had a job were more likely to use skilled ANC services (AOR = 1.1, 95% CI = 1.1-1.3).

##### 

**Household wealth index** Household wealth index was significantly associated with the use of all the three maternal health services. Women from wealthier households used skilled ANC, delivery and PNC services at significantly higher rates than those women from less wealthy households. Women from the middle wealth households (AOR = 1.1, 95% CI = 1.2-1.8) and women from the richest households (AOR = 1.1, 95% CI = 2.9-4.8) were more likely to use ANC service compared with those who were from the poorest wealth quintile. Regarding the use of skilled delivery attendants and PNC services, women from the richest households were more likely to use skilled delivery attendants (AOR = 3.0, 95%CI = 2.1-4.2) and PNC services (AOR = 2.4, 95% CI = 1.7-3.5) than those who were from the poorest households.

## Discussion

Health care seeking may be influenced by the cultural backgrounds, beliefs, norms and values of specific ethnic groups and religion. Ethnicity and religion are often thought to influence beliefs, norms and values in relation to pregnancy, childbirth and utilization of services [13,15]. In this study, Christian and Muslim women were more likely to use maternal health services than traditional and other religions. This result is consistent with other studies. This may be because women with traditional religion may be less modern and more inclined to traditional beliefs. Regarding ethnicity, Wolaita ethnic groups were less likely to use skilled ANC and delivery care than other ethnic groups. The reason for the low maternal health service utilization by the Wolaita ethnic group may be due to the fact that these ethnic groups culturally may not support facility delivery due to their cultural beliefs and values on maternal health care, and this needs further qualitative study to explore the detailed reasons. A study by Shiferaw and colleagues identified that one of the most important reasons for not seeking institutional delivery in Ethiopia was the belief that it is not necessary and not customary [16]. A study in Vietnam also showed that the risk of not giving birth in a health facility increased significantly among ethnic minority women living in rural areas [17]. Further qualitative investigation on the effects of cultural practices is required. Studies done in developing countries showed that maternal health services utilization is affected by ethnicity, culture and religion of women. This was explained by the fact that women’s autonomy, gender relationships and social networks are affected by ethnicity, culture and religion [18,19].

This study has found that education of women and their husbands has had a significant effect on the utilization of all the three maternal health services. The effect of maternal education level was stronger than husband’s education. These results have been consistently supported by many other studies which showed a positive influence of education on maternal health service utilization [14,20-22]. This positive relationship may be explained by the fact that educated women are more knowledgeable on the importance of maternal health services; they may have access to written information and may have a more modern cultural perspective. Educated husbands may have a better communication with their wives and willingness to discuss the use of maternal health services. They may also provide more autonomy to their wives [21,23-25].

Physical accessibility is one of the most important variables in health service utilization. Several studies have identified that physical proximity of health care services plays an important role in service utilization. In this study, urban residence was significantly association with the utilization of ANC, delivery and PNC services. This result has been consistent with many other studies [14-16,18,23,26]. The difference may be due to the increased availability of infrastructure (shorter distance to health facilities, better roads and transportation) in urban areas than rural areas. In Ethiopia, there is a significant difference in the availability of health workers among regions. Addis Ababa and Dire Dawa city administrations with an urban population proportion of 100% and 67.5% respectively have a higher number of medical doctors than other regions that have a more rural population. Addis Ababa and Dire Dawa city administrations have one medical doctor for 3,056 and 6,796 respectively. Other regions like Amhara and Oromia, with an urban population proportion of 12.6% and 12.2% respectively have very few doctors compared with the other regions [27]. This shows that urban areas are more advantaged in terms of accessing health professionals than rural areas.

Other similar studies identified residence as a factor for maternal health service utilization. The studies showed that maternal health service utilization is higher among urban residents than rural residents [16,22,26,28]. A study done in Ethiopia on why women choose to deliver at home showed that lack of transportation was one of the major factors [16].

This study has found that household wealth status is significantly associated with the utilization of all the three maternal health services. Women who are from a household with a higher wealth quintile are more likely to utilize all the maternal health services than those who are from the poor wealth households. This result is consistent with other similar studies [21,29]. This is expected since access to health services utilization in Ethiopia mainly depends on out of pocket payment [30]. Though the services for ANC, delivery and PNC are exempted, women are expected to pay for medications and additional transportation costs contribute to the high cost of seeking care and may deter women from utilizing services. This has also been revealed in other similar studies [21]. A similar study in Ghana has revealed that wealth has a significant influence on the use of skilled delivery attendants. It showed that the odds of having a skilled attendant at delivery for women in the poorest wealth quintile are 94% lower than that for women in the highest wealth quintile and almost 5 times higher for women with completed primary education relative to those less educated [29].

This study has shown that parity determines health service utilization more than maternal age. Women who had only one birth were more likely to use skilled delivery attendants and PNC services. This may be due to the fact that women with higher parity may have developed self-confidence to deliver at home and may not be motivated to use a health professional. Moreover, primipara women may be afraid of pregnancy complications and outcomes since they have had no prior delivery experience. This may be because of the perceived risk of first pregnancy on health. A study in Bangladesh has shown a similar result which found that a woman is more likely to seek maternal health care services for first order than higher-order births because of perceived risk associated with first pregnancy [31]. A study in Kenya also showed that women of high parity are less likely to initiate ANC on time or to make the recommended number of visits, assuming that they are experienced [28]. The other reason may be because of prior bad health facility experiences. Having more children may also cause resource constraints, which have a negative effect on health care utilization [31]. Many studies have shown consistent finding on the low likelihood of having a health facility delivery as number of children ever born increased [32,33].

Decision making power of women can have a significant effect on the ability of women to seek health services and/or contribute to delays in accessing and receiving medical care even in places where services are readily available [21]. In this study, women who have been able to decide on health care spending by themselves were more likely than women whose health care spending was controlled by other people. This may be because if resources are controlled by others, women do not have the freedom to use services whenever they need care. Autonomy may also be associated with other variables like with education of women and urban residence, both of which are factors that increase the likelihood of the use of maternal health services.

The use of ANC care during pregnancy was found to significantly affect the use of skilled delivery attendants. This may be because women will be aware of the importance of attending delivery in health facilities as they might be educated during the ANC session. Many other studies have found a similar result [22,34]. This result shows that use of ANC is one of the strongest determinants for the use of skilled delivery attendants during delivery and PNC services after delivery.

Women who are working earn money so that they can have the economic ability to pay for health services. However, work status of women was found to be associated with the use of ANC services only. It is not associated with the use of delivery and PNC services. The reason for the absence of association may be explained because even though they are working, the decision on health spending may be made by their husbands.

Use of public media sources like listening radio, watching television and reading newspapers increases the awareness of people on health and other matters. In this study, use of these public media sources significantly affected the use of ANC services. However, the association is not consistent with the use of delivery attendants and PNC services, which may be due to the fact that the majority of women are illiterate and live in rural areas where television, radio and newspapers are not available. Therefore, this may be due to the low proportion of households who have radio and television in Ethiopia. A study in Nigeria has shown that community media saturation was found to be a strong predictor of maternal health service utilization [35].

## Conclusion

This study has examined the determinants of ANC, use of skilled delivery attendants and PNC services in Ethiopia. It shows that utilization of maternal health services in Ethiopia is very low and is affected by a number of socio-cultural, perceived benefits and accessibility-related factors.

Education level of women and their husbands is one of the strongest determinants of the use of maternal health services. Service utilization increased consistently as the education level of women and their husbands increase. There is a high inequality in service utilization between urban and rural areas. Household wealth and level of autonomy of women on health spending are important determinants of service utilization. Ethnicity is also a determinant factor of service utilization, which shows the importance of giving a due attention to the empowerment of women from less advantaged ethnic groups. Use of ANC during pregnancy is a major predicator of subsequent use of skilled delivery and PNC services, which shows the importance of ANC as an important entry point to increase the low utilization of skilled delivery and PNC services.

The findings of the study show the importance of women education, empowerment and economic improvement for women. Moreover, infrastructural improvements like improving roads and providing transportation services for pregnant women in rural areas is recommended.

In general, the study findings show that the determinants of maternal health service utilization are multi-sectoral signifying a multi-sectoral approach to tackle it. The health, education, social service, agriculture, transportation, employment, and other sectors should be involved for long term improvement in service access and utilization. Further study need to be done on health facility related factors, which are the major causes of the third delay in maternal mortality.

## Competing interests

The authors have no financial and non-financial competing interests.

## Authors’ contribution

SMT: Conceptualized the design and overall study, wrote the result, made the analysis and interpretation, and wrote the result, discussion and conclusion sections. LSL: Wrote sections of the manuscript and led the literature review part. Participated in analysis and reviewed the final manuscript. VG: Participated in literature review and data analysis, wrote the results section and reviewed draft of the manuscript. All authors read and approved the final manuscript.

## Authors’ information

SMT: - Graduate of Master of Public Health (MPH) in reproductive health in 2007 from Addis Ababa University and also graduated with European masters in Sustainable Regional Health Systems in 2013 from Vilnius University and currently working as regional health informatics coordinator at Tulane International in Addis Ababa, Ethiopia.

LSL: - A professor at the University of Central Florida and also a managing director of Lieberman consulting in Florida, USA. Experienced researcher in biomedical Anthropology, Nutrition and Public Health

VG: - A professor at Vilnius University, Lithuania. Also a coordinator of RegHealth Master Program of European Commission Experienced in researches related with economics and Public health.

## Pre-publication history

The pre-publication history for this paper can be accessed here:

http://www.biomedcentral.com/1471-2393/14/161/prepub

## References

[B1] World Health Organization WHO 2012 maternal and child health fact sheethttp://www.who.int/mediacentre/factsheets/fs348/en/

[B2] UNFPA 2013 fact sheethttp://www.unfpa.org/public/home/mothers/pid/4381

[B3] UN The Millennium Development Goals Report 2012 2012 New York: United Nations

[B4] UN and African Union Commision Report on Progress in Achieving the Millennium Development Goals in Africa, 2013 2013 Abidjan: Côte d'Ivoire E/ECA/COE/32/3. Retrieved April 10, 2013, from http://www.uneca.org/sites/default/files/document_files/report-on-progress-in-achieving-the-mdgs-in-africa.pdf

[B5] WHO, UNFPA, UNICEF and World bank Trends in maternal mortality: 1990-2010 2012 Geneva: WHO, UNICEF, UNFPA and The World Bank estimates

[B6] CSA [Ethiopia] and ORC Macro Ethiopia Demographic and Health Survey 2000 2001 Addis Ababa, Ethiopia and Calverton, Maryland, USA: Central statistical Agency and ICF International

[B7] CSA [Ethiopia] and ORC Macro Ethiopian Demographic and Health Survey 2005 2006 Addis Ababa, Ethiopia and Calverton, Maryland, USA: Central statistical Agency and ICF International

[B8] CSA [Ethiopia] and ICF International Ethiopia Demographic and Health Survey 2011 2012 Addis Ababa Ethiopia and Calverton, Maryland, USA: Central statistical Agency and ICF International

[B9] Carroli G Rooney C Villar J How effective is antenatal care in preventing maternal mortality and serious morbidity? An overview of the evidence Paediatr Perinat Epidemiology 2001 15 suppl 1 1 42 10.1046/j.1365-3016.2001.0150s1001.x 11243499

[B10] Wagstaff A Claeson M The Millennium Development Goals for Health: Rising to the Challenges 2004 Washington, DC: The World Bank

[B11] USAID Measure DHS: Demographic and Health Surveys 2011 Retrieved from Measure DHS: http://dhsprogram.com/Data/

[B12] Levy Lemeshow Sampling of Populations 2001 New York: John Wiley and Sons

[B13] Andersen R Newman JF Societal and Individual Determinants of Medical Care Utilization in the United States Milbank Q 2005 83 4 1 28 Retrieved from http://www.milbank.org/uploads/documents/QuarterlyCentennialEdition/Societal%20and%20Indv.pdf 4198894

[B14] Thadeus Maine Too far to walk: Maternal mortality in context Soc Sci Med 1994 38 8 1109 1120 10.1016/0277-9536(94)90226-7 8042057

[B15] Gabrysch S Campbel O Still too far to walk: Literature review of the determinants of delivery service use BMC Pregnancy Childbirth 2009 9 34 10.1186/1471-2393-9-34 19671156 PMC2744662

[B16] Shiferaw S Spigt M Godefrooij M Melkamu Y Tekie M Why do women prefer home births in Ethiopia? BMC Pregnancy Childbirth 2013 13 5 10.1186/1471-2393-13-5 23324550 PMC3562506

[B17] Målqvist M Lincetto O Du NH Burgess C Hoa DTP Maternal health care utilization in Viet Nam: increasing ethnic inequity Bull World Health Organ 2013 91 254 261 10.2471/BLT.12.112425 23599548 PMC3629455

[B18] Say L Raine R A systematic review of inequalities in the use of maternal health care in developing countries: examining the scale of the problem and the importance of context Bull World Health Organ 2007 85 812 819 10.2471/BLT.06.035659 18038064 PMC2636485

[B19] Singh P Rai R Alagarajan M Singh L Determinants of Maternity Care Services Utilization among Married Adolescents in Rural India PLoS One 2012 7 2 e31666 DOI: 10.1371/journal.pone.0031666 10.1371/journal.pone.0031666 22355386 PMC3280328

[B20] Bell J Curtis SL Alayón S Trends in Delivery Care in six Countries. DHS Analytical Studies No. 7 2003 Calverton, Maryland: ORC Macro and International Research Partnership for Skilled Attendance for Everyone (SAFE)

[B21] Ahmed S Andreea A Creanga mail Gillespie DG Tsui AO Economic Status, Education and Empowerment: Implications for Maternal Health Service Utilization in Developing Countries PLoS One 2010 5 6 e11190 doi:10.1371/journal.pone.0011190 10.1371/journal.pone.0011190 20585646 PMC2890410

[B22] Mengesha ZB Biks GA Ayele TA Tessema GA Koye DN Determinants of skilled attendance for delivery in Northwest Ethiopia: a community based nested case control study BMC Public Health 2013 13 130 10.1186/1471-2458-13-130 23402542 PMC3577480

[B23] Sharma S Sawangdee Y Sirirassamee B Access to health: women’s status and utilization of maternal health services in Nepal J Biosoc Sci 2007 39 5 671 692 10.1017/S0021932007001952 17359562

[B24] Anyait A Mukanga D Oundo B Nuwaha F Predictors for health facility delivery in Busia district of Uganda: A cross sectional study BMC Pregnancy and child birth 2012 12 132 10.1186/1471-2393-12-132 PMC3514288 23167791

[B25] Cham M Sundby J Vangen S Maternal mortality in the rural Gambia, a qualitative study on access to emergency obstetric care Biomed Central: Reprod Health 2005 2 3 10.1186/1742-4755-2-3 PMC1142340 15871743

[B26] Stephenson R Matthews Z Maternal health care service use among rural-urban migrants in Mumbai, India Asia Pac Popul J 2004 19 1 39 60

[B27] CSA [Ethiopia] Summary and Statistical Report of the 2007 Population and Housing Census 2008 Addis Ababa: Federal Democratic Republic of Ethiopia Population and Census Commission

[B28] Fotso JC Ezeh A Oronje R Provision and Use of Maternal Health Services among Urban Poor Women in Kenya: What Do We Know and What Can We Do? J Urban Health 2008 85 3 428 442 10.1007/s11524-008-9263-1 18389376 PMC2329740

[B29] Arthur E Wealth and antenatal care use: implications for maternal health care utilisation in Ghana Health Econ Rev 2012 2 14 10.1186/2191-1991-2-14 22866869 PMC3484029

[B30] Ministry of Health of Ethiopia Ethiopia's Fourth National Health Accounts, 2007/2008 2010 Addis Ababa: Ministry of Health [Ethiopia]

[B31] Chakraborty N Ataharul Islam M Islam Chowdhury R Bari W Hanumakhter H Determinants of the use of maternal health services in rural Bangladish Health Promot Int 2003 18 4 327 10.1093/heapro/dag414 14695364

[B32] Mekonnen Y Mekonnen A Factors influencing the use of maternal healthcare services in Ethiopia J Health Popul Nutr 2003 21 4 374 382 15038593

[B33] Eijk V Bles H Odhiambo F Ayisi J Blokland I Rosen D Use of antenatal services and delivery care among women in rural western Kenya: a community based survey Reprod Health 2006 3 2 10.1186/1742-4755-3-2 16597344 PMC1459114

[B34] Abebe F Berhane Y Girma B Factors associated with home delivery in Bahirdar, Ethiopia: A case control study BMC Res Notes 2012 5 653 10.1186/1756-0500-5-653 23176369 PMC3554461

[B35] Babalola S Fatusi A Determinants of use of maternal health services in Nigeria - looking beyond individual and household factors BMC Pregnancy Childbirth 2009 9 43 10.1186/1471-2393-9-43 19754941 PMC2754433

